# Preparation and Characterization of Zeolite/TiO_2_ Cement-Based Composites with Excellent Photocatalytic Performance

**DOI:** 10.3390/ma11122485

**Published:** 2018-12-07

**Authors:** Gang Liao, Wu Yao, Junqing Zuo

**Affiliations:** 1 Department of Transportation and Municipal Engineering, Sichuan College of Architectural Technology, Chengdu 610399, China; gangliao@vip.163.com; 2 Key Laboratory of Advanced Civil Engineering Materials (Tongji University), Ministry of Education, Shanghai 201804, China; 3 Shanghai Construction Group Co., Ltd., Shanghai 200080, China; junqingzuo@163.com

**Keywords:** Zeolite/TiO_2_ composite, photocatalytic cement-based material, photocatalytic efficiency

## Abstract

A zeolite/TiO_2_ composite (ZTC) was applied to prepare a high-quality photocatalytic cement-based material (PCM). The acetone degradation experiment and micro measurements including XRD(X-Ray Diffractometer), SEM-EDS(Scanning Electron Microscope-Energy Dispersive Spectrometer), BET(BET Specific Surface Area Tester), FTIR(Fourier Transform Infrared Spectrometer) were conducted to characterize the photocatalytic efficiency and physicochemical properties of PCM, respectively. Results show that TiO_2_ particles disperse on the surface of a PCM homogeneously and provide abundant active sites for photocatalytic reactions. Compared to a normal photocatalytic cement-based material (NPCM), the TiO_2_ content of a PCM is lower and its photocatalytic efficiency is higher. The ZTC frees TiO_2_ particles from the impacts of cement hydration products and increases the adsorption volume of acetone. The photocatalytic performance of the PCM was stable after repeated tests. Using the ZTC as a photocatalyst has a prominent effect on the photocatalytic performance of the PCM.

## 1. Introduction

Urban areas are facing serious air pollution because of substantive industrial emissions, massive automobile exhaust, and more urban construction. Air pollution is a great threat to human health. In general, volatile organic compounds (VOCs) and nitric oxides (NO_x_) are considered to be the major pollutants [1,2,3]. Photocatalytic oxidation is an efficient and cost-effective technology to eliminate pollution. In addition, TiO_2_ is a typical photocatalytic material known for its excellent photodegradation [4,5,6,7,8,9]. This cement-based material is widely used as the fundamental construction material around the world. In recent years, the use of TiO_2_ (and other building materials) for air pollution control has attracted worldwide attention. Many investigations have been conducted and some progress has been made [10,11,12,13,14].

However, photocatalysis is a complex surface phenomenon, and its reaction rate depends on many factors. The availability of active sites and the adsorption properties of photocatalysts govern the reaction process of gaseous photocatalytic oxidation [15]. It is easy to combine TiO_2_ with a cement matrix by coating, spraying, and mixing, but some intrinsic properties of cement-based material, e.g., low porosity, small specific surface area, complex chemical environment, restrict the photocatalytic activity of TiO_2_ and weaken its long-term performance. Recently, more studies have been dedicated to the performances of cementitious materials when mixed with photocatalytic titanium dioxide. Folli et al. [16] reported that when nano-TiO_2_ was added to cement, it was inclined to a strong agglomeration of nano-TiO_2_, thereby decreasing the specific surface area and photocatalytic activity. Lackhoff et al. [17] pointed out that the carbonation of the TiO_2_-modified cements led to a noticeable loss in catalytic efficiency because of the changes in the cement surface structure. The research of Chen et al. [18] showed that when nano-TiO_2_ was embedded in cement-based materials, the latter became potential nucleation sites for cement hydration products. When these hydration reactions took place, the hydration products gradually bonded the individual nano-TiO_2_ particles together, forming a dense coating on the TiO_2_ surface, thus significantly diminishing its photocatalytic activity. Neppolian et al. [19] investigated the influence of pH on its photocatalytic effect, and the results indicated that high pH values (pH > 10) could decrease the removal rate of reactive yellow 17 dye. Moreover, Vallée et al. [20] found that the transition metal in the cement pastes incurred the electron-hole recombination, thus deactivating the photocatalysts. Lee et al. [21] demonstrated that the specific surface area of cement-based materials can be tailored to enhance the adsorption capacity, but the effect was not significant. In summary, the problems can be grouped into the 4 categories: (1) the influence of the ions (including Na^+^, Ca^2+^, OH^−^, etc.); (2) the agglomeration of nano-TiO_2_ and the coverage of TiO_2_ by a cement hydration product; (3) low diffusion and adsorption of the pollutant; (4) subsequent peeling of TiO_2_. Although some innovative approaches have been performed [11,12,22], the problems described above have unfortunately not been resolved properly. In the field of catalysts, natural zeolite (NZ) was an optimal carrier for photocatalysts due to its high specific surface area, high adsorption capacity, and high stability [23,24]. The Zeolite/TiO_2_ composite was extensively investigated in the field of photocatalytic pollution mitigation. Jansson I. et al. [25] reported that the incipient wet impregnation method was used to prepare Zeolite/TiO_2_ composite, which led to a significant photodegradation of HCHO with a maximum conversion value of around 80%. The Nano-TiO_2_ particles homogeneously distributed on zeolitic structures. Sun Q et al. [26] found that the crystalline phase, crystallite size, specific surface area, pore structures, adsorption, and photocatalysis ability of the Zeolite/TiO_2_ highly depended on the calcination temperature. If a Zeolite/TiO_2_ composite is applied to the photocatalytic cement-based material (PCM), there are several advantages: (1) excellent adsorption properties lead to higher concentration of contaminant around TiO_2_, which will facilitate the reaction; (2) high porosity and large specific surface area make it facile for dispersion and immobilization of TiO_2_ on the surface of carrier; (3) the middle-carrier will prevent TiO_2_ particles from the impacts of cement hydration production. It is a promising way to enhance the photocatalytic efficiency of PCMs by introducing a Zeolite/TiO_2_ composite.

In this work, the aim is to enhance the photocatalytic efficiency of PCMs by making TiO_2_ particles disperse homogeneously, thereby improving the adsorbent properties of the matrix and protecting the TiO_2_ from the influence of the cementitious system. The main procedures of the experiment are described below. Firstly, the natural zeolite was modified via alkali activation and calcination to obtain the modified zeolite (MZ). The TiO_2_ sol was made through the hydrolysis of tetrabutyl titanate (TBOT). Subsequently, the MZ was mixed with TiO_2_ sol to prepare the Zeolite/TiO_2_ composite (ZTC). Following this, the ZTC was sprayed on the surface of cement substrate to prepare the PCM. Lastly, XRD, BET, SEM-EDS, FTIR and GC-FID(Gas Chromatography-Flame Ionization Detector) were used to characterize the properties and the photocatalytic performance of the PCM.

## 2. Materials and Method

### 2.1. Materials

The natural zeolite in the form of clinoptilolite was purchased in Gongyi, Henan Province, China. The physical characteristics and chemical composition of the zeolite are shown in Table 1 and Table 2, respectively. The cementitious material was Ordinary Portland Cement 42.5 produced by Gezhouba Cement Co., Ltd., Jingmen, China. The tetrabutyl titanate (TBOT) was used as the TiO_2_ precursor, obtained from Sinopharm Chemical Reagent Co., Ltd., Shanghai, China. Acetone was chosen as the gaseous pollutant.

### 2.2. Preparation of Zeolite/TiO_2_ Composite (ZTC)

The Zeolite/TiO_2_ composite (ZTC) was synthesized as follows: 20 g of natural zeolite (NZ) was washed to eliminate the mud and then activated for 6 h with a NaOH aqueous solution (200 mL, 1.5 mol/L). After alkali activation, the treated zeolite was washed with deionized water until the pH value was 7 or 8 and subsequently thermally treated at 400 °C for 2 h to remove the water and other impurities. After washing and calcination, the modified zeolite (MZ) was obtained. The TiO_2_ sol was synthesized by modifying the preparation described by Yang et al. and Yu et al. [27,28]. Solution A (made up of 31.96 g of TBOT and 47.94 g of absolute ethyl alcohol) and solution B (made up of 319.6 g of deionized water, 47.94 g of absolute ethyl alcohol and 2.56 g of hydrogen nitrate) were prepared respectively. Solution A was added into solution B at the speed of 1–2 drops per second while stirring. In addition, the hydrolysis reaction {Ti(O-CH_4_)_4_ + 4H_2_O → Ti(OH)_4_ + 4C_4_H_9_OH} took place at the same time. The mixed solution was then heated in a water bath at 50 °C for 12 h until complete peptisation. In the heating procedure, a white slurry appeared and gradually turned into a kind of nattier transparent blue solution: this was the TiO_2_ sol, and the content of TiO_2_ in the sol was about 2.12% wt. A certain mass of TiO_2_-sol and MZ were mixed together, thereafter the mixture was treated with stirring and ultrasonification for 30 min, and then kept in a negative pressure still of 0.5 MPa for 2 h. The treated mixture was dried at 105 °C for 2 h to remove the water, absolute ethyl alcohol, TBOT, and other residua. Finally, the dried mixture was calcined at 400 °C for 2 h to obtain the ZTC. A total of three groups of zeolite/TiO_2_ composite samples were formulated, labeled as ZTC-1 (sol/MZ mass ratio of 0.47), ZTC-2 (sol/MZ mass ratio of 2.36), and ZTC-3 (sol/MZ mass ratio of 4.72). A certain amount of the TiO_2_-sol was dried at 105 °C for 2 h and then calcined at 400 °C for 2 h to obtain the TiO_2_ powder, called as HTOP. The TiO_2_ content of all samples are shown in Table 3.

### 2.3. Preparation of Photocatalytic Cement-Based Material (PCM)

The cement matrix (Ø50 × 5 mm) was produced as follow: 22 g of cement was mixed with 9.9 g of water, and the cement paste was cast into special mould for 30 min. Before the solidification of the cement paste, 0.22 g of the ZTC was sprayed onto the surface of the paste, and all samples were cured in a standard curing chamber at 25 ± 1 °C and 60 ± 5% RH (relative humidity) for 1 day. After demoulding, the samples were dried at 105 °C, and then the PCM was ready. A total of four kinds of PCM samples were prepared, denoted as PCM-n (n = 0, 1, 2, 3). For comparison, normal photocatalytic cement-based materials (NPCM) were fabricated by incorporating HTOP, named as NPCM-n (n = 1, 2, 3). The compositions of all samples are presented in Table 4.

### 2.4. Physicochemical Properties Characterization

The crystalline phases of the samples were identified with an X-ray diffraction detector (Rigaku D/max2550, Tokyo, Japan) with a Cu Ka ray source operating at 40 kV and 100 mA. The diffraction angles (2θ) between 5° and 70° were continuously recorded with the interval of 0.02° at the speed of 4°min^−1^. The ZTC were grinded to a micron-sized powder and plated with a layer of gold film. The surface morphology and microstructure of the ZTC were observed by SEM (FEI Quanta 200F, Hillsboro, America). In addition, the micro-area element was analyzed by EDS. The specific surface area and pore size distribution of the ZTC were revealed by N_2_ adsorption-desorption isotherms which were measured by the instrument (Beishide 3H-2000PS2, Beijing, China). The infrared spectrum was measured by FTIR (BRUKER EQUINOX55, Karlsruhe, Germany) to verify if there is a chemical bond between TiO_2_ and zeolite.

### 2.5. Evaluation of Photocatalytic-Degradation Performance

**Figure 1:**
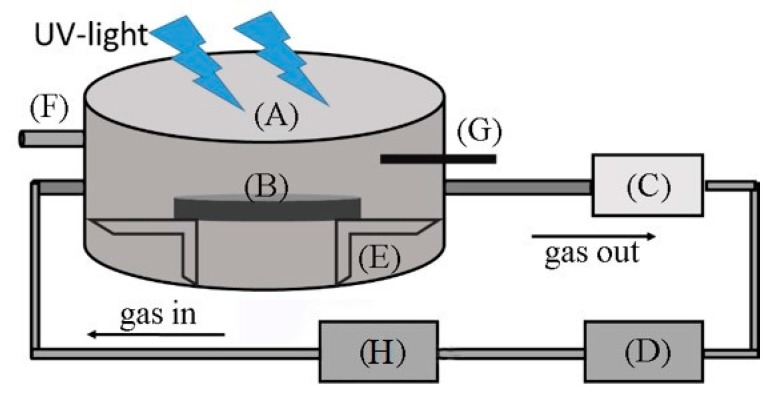
Schematic diagram of the gas-phase photocatalytic test device: (**A**) stainless steel reactor; (**B**) specimen; (**C**) vacuum pump; (**D**) gas chromatograph and computer system; (**E**) sample holder; (**F**) reactant injection pore; (**G**) temperature and humidity sensor; (**H**) H_2_O vaporizer.

The photocatalytic degradation efficiency was measured by a home-made gas-phase photocatalysis test device. The device consisted of a stainless-steel reactor, a gas conduit, an ultraviolet (UV)-lamp, a gas chromatograph, a vacuum pump, an H_2_O vaporizer, and temperature and humidity sensor, as shown in Figure 1. The reactor was a hermetically sealed stainless-steel cylinder (3.61 L). The cover of the reactor was made of quartz glass with a high light transmittance. The gas was driven to cycle in the device by a vacuum pump (flow rate of 0.4 Lmin^−1^). The light source was a high-pressure Hg lamp with a wavelength of 365 nm, thus belonging to the UV-light spectrum (10~400 nm). The intensity of the UV-light in the experiment was measured with a UV-A radiometer (LUTRON, UV-340-A, Taiwan). The intensity of the light in the experiment was about 1.2 mW/cm^2^, which was equal to the light level of central China, where the UV light intensity is about 1.0–3.0 and 1.5 mW/cm^2^ in summer daylight and winter daylight, respectively [29]. To reach the intensity (1.2 mW/cm^2^), the distance between the lamp and the reactor was adjusted to 15 cm.

Exactly 1 μL of liquid acetone was injected into the reactor and was kept for 30 min without light to guarantee complete evaporation. The initial concentration of gaseous acetone was about 3.76 μmol/L in the reactor. The temperature of the reactor was maintained at 37 °C and the relative humidity (RH) was 20% [30]. Once the irradiation of the UV-light occurred, the photocatalysis reactions started immediately. In the reaction process, the concentration of gaseous acetone was measured by the gas chromatograph every 5 min during the reaction process. When one test was finished, the just tested specimen was heated in an oven for 2 h at 70 °C to be acetone-free, and the reactor was wiped with absolute ethyl alcohol and irradiated with a UV-lamp for 30 min. The degradation rate of the acetone was calculated using Formula (1), and the reaction rate were calculated using Formula (2),

(1) $$w\%=\frac{C}{C_{0}}$$

(2) $$ln^{\frac{c}{c_{0}}}=-k\cdot t$$

where w% was the degradation rate of acetone, *C*_0_ and *C* represented the initial and final concentration of acetone respectively, *k* was the reaction rate, and *t* was the reaction time.

## 3. Results and Discussion

### 3.1. Physicochemical Properties

**Figure 2:**
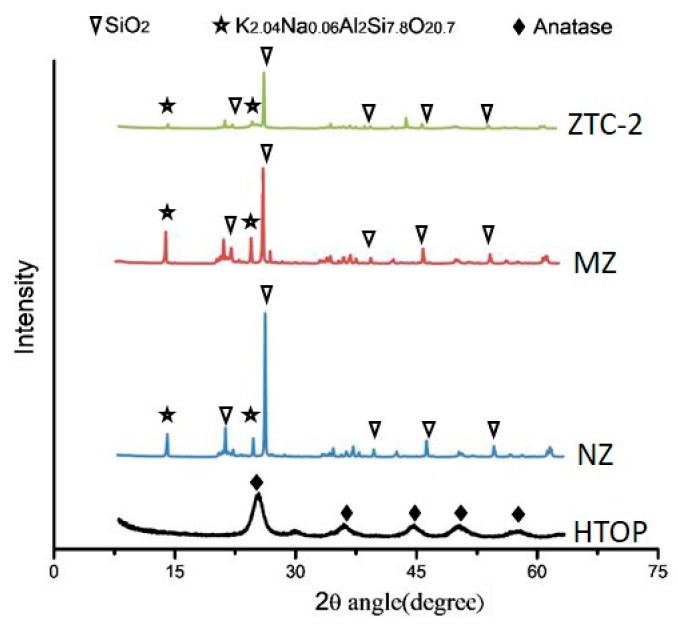
X-ray diffraction patterns of HTOP, NZ, MZ, and ZTC-2.

The XRD results are shown in Figure 2. The diffraction peaks of TiO_2_ in form of anatase were observed in the diffractogram of HTOP. Calculated using the Scherrer equation, the particle size of HTOP was about 10 nm. It is well-known that nano-TiO_2_, particularly in the anatase form, exhibits a photocatalytic activity under UV irradiation. It was inferred that the ZTC based on HTOP had the potential for good photocatalytic performance. The intensity of the characteristic peaks of the MZ decreased slightly after alkali activation and calcination. It was mostly because the Si embedded in the zeolite framework was selectively dissolved and the chemical-bonding water was destroyed. The diffractogram of ZTC-2 also shows the characteristic diffraction peaks of the MZ, but the characteristic peaks intensity of ZTC-2 is weaker than the MZ. This dilution effect was more evident for the TiO_2_ crystal phases. In fact, the characteristic peak (2θ = 25.3°) of TiO_2_ was not detected in the ZTC, and it may be owing to the smaller content of TiO_2_. On the other hand, the absence of characteristic peaks for TiO_2_ indicated that TiO_2_ particles were well dispersed on the zeolite, which in fact prevented the growing of large TiO_2_ crystallites [25]. 

**Figure 3:**
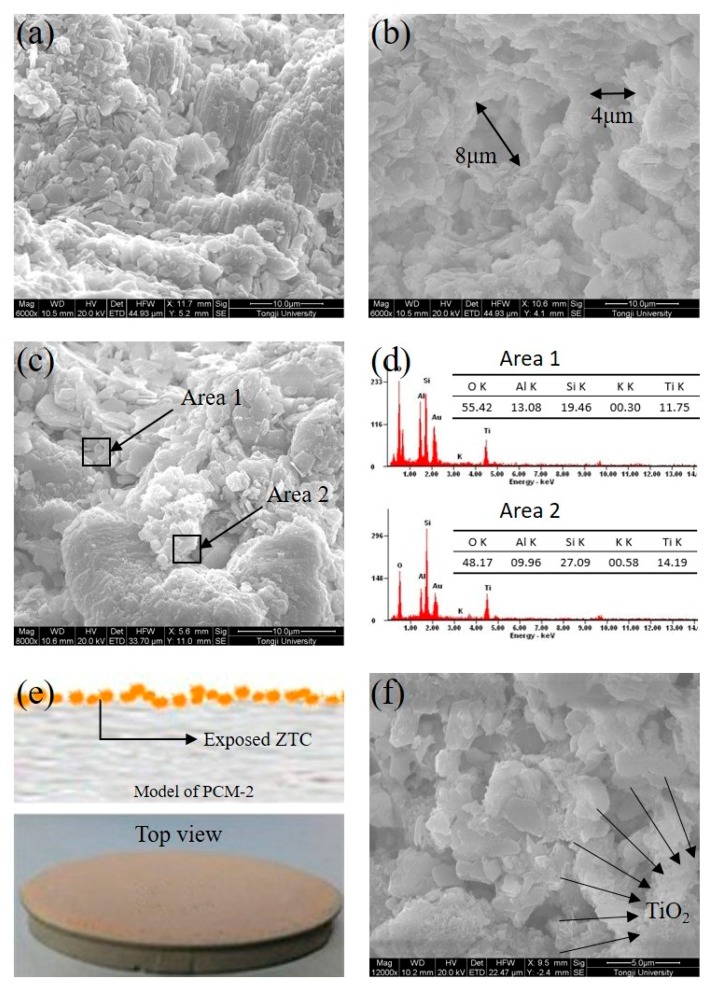
Surface morphology and EDS analysis: (**a**) SEM image of NZ; (**b**) SEM image of MZ; (**c**,**d**) SEM image of ZTC-2 with EDS; (**e**) photograph of the surface layer of PCM-2; (**f**) SEM image of exposed ZTC-2.

SEM images and EDS analysis are shown in Figure 3, and the microstructure of samples is revealed. Compared with NZ (Figure 3a), the surface of the MZ (Figure 3b) was rougher, and more micropores (about 6 μm) were observed on the surface, which benefited the deposition of nano-TiO_2_ and adsorption of pollutant. As shown in Figure 3c, Area 1 and Area 2 were randomly selected for the micro-area element analysis. In addition, the EDS results (Figure 3d) showed that the elements (including Si, O, Al, K, Ti) were detected, among which, the content of Ti was considerable. Based on the results of EDS, it was verified that TiO_2_ had already precipitated on the surface of zeolite. Considering the 2 microregions were selected randomly and the content of Ti in Area 1 (11.75%) was close to Area 2 (14.19%), it can be inferred that the nano-TiO_2_ particles were homogeneously distributed on the surface of the MZ within a certain range. Figure 3e shows the top view photograph of the surface layer of PCM-2. The orange ZTC-2 particles were immobilized on the surface layer of PCM-2, and a little dark gray cement paste was observed. One could argue that ZTC-2 was adequately exposed to the air at the macro-scale. To better understand the immobilization of ZTC-2 on the surface of PCM-2, the model of PCM-2 is also shown in Figure 3e. As shown in Figure 3f, the cement hydration product could not be observed except for TiO_2_ film. It was indicated that the exposed ZTC-2 was not covered by the cement hydration product, and the adverse influence of the cement ion species as well as carbonization were weakened, which thus helped enhance the photocatalytic property. This contribution was related to the separation between the zeolite and the cement.

**Figure 4:**
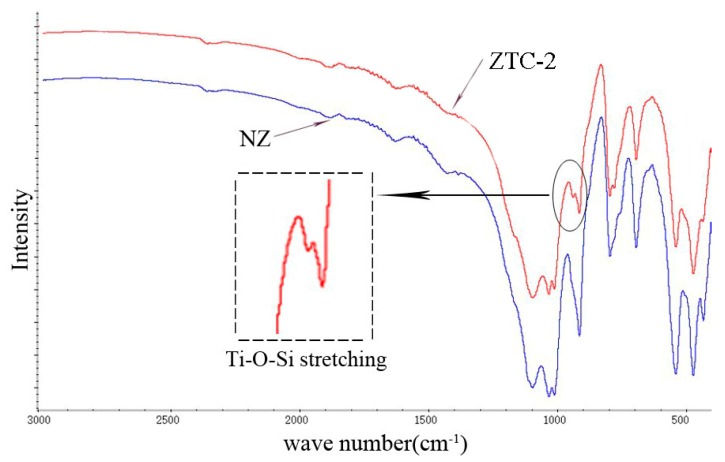
Infrared spectrum of NZ and ZTC-2.

The chemical changes during the process of the ZTC synthesis were monitored by FTIR, and the IR spectra are shown in Figure 4. For the NZ sample, peaks were observed at the range of 440~600 cm^−1^ and 700~800 cm^−1^, which was the characteristic of Si-O bond stretching vibration. The IR spectra of ZTC-2 was almost similar to the NZ with the exception of a vibrational band at 960 cm^−1^, which attributed to the antisymmetric Ti-O-Si stretching modes of a corner-sharing tetrahedral [31]. It was inferred that there was chemical bond between TiO_2_ and the zeolite. Under the force of the chemical bond, TiO_2_ particles were firmly fixed on the surface of the ZTC. This effect was significant for the long-term performance of the PCM.

**Figure 5:**
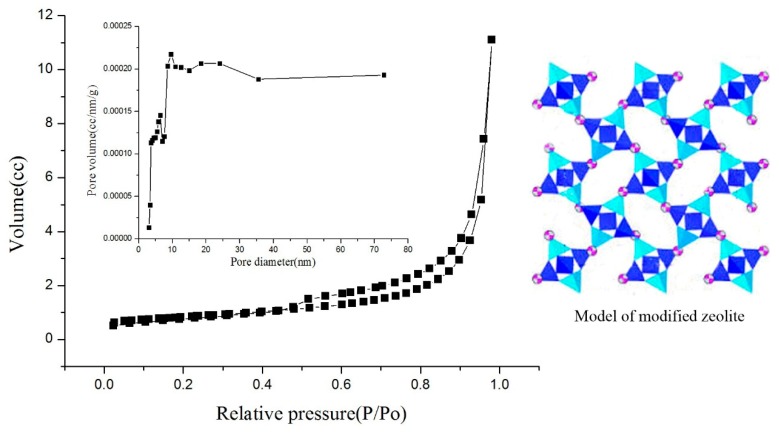
N_2_ adsorption–desorption isotherms and pore size distribution of MZ.

**Figure 6:**
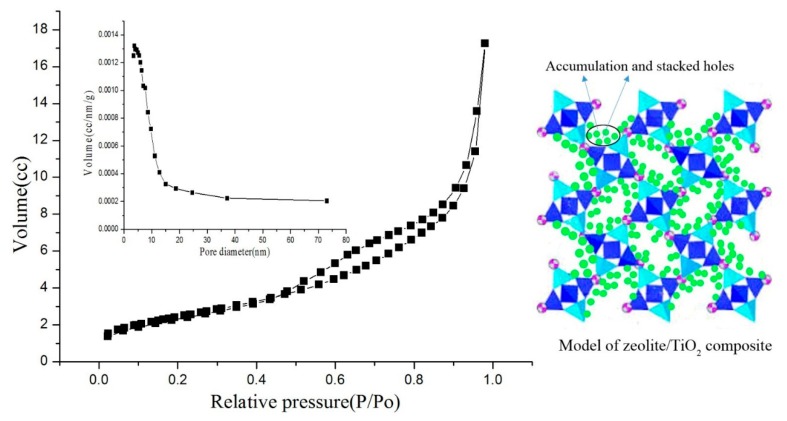
N_2_ adsorption–desorption isotherms and pore size distribution of ZTC-2.

The nitrogen adsorption–desorption isotherms of the MZ and ZTC are presented in Figure 5 and Figure 6. The isotherms were of type IV according to BDDT (Brunauer, Deming, Deming and Teller) classification. A hysteresis loop could be observed at relative pressure between 0.48 and 1, indicating that there were mesoporous structures [11,32]. As shown in Figure 5 and Figure 6, the isotherms of the MZ were different from ZTC-2, which indicated that after TiO_2_ was loaded onto the surface of the MZ, the specific surface area was changed. From Table 5, the results showed that ZTC-2 recorded a slight reduction of BET specific surface area and total pore volume as compared to that of the MZ, probably due to the pore blocking by the TiO_2_ particles. The pore size distribution curve of the MZ was different from ZTC-2, and the most probable aperture was changed from 13 nm (MZ) to 5 nm (ZTC-2). This can be explained by the fact that the nano-TiO_2_ particles were pressed into the pores of zeolite under negative-pressure, which led to more stacked holes. However, the BET specific surface area of ZTC-2 still maintained 319 m^2^g^−1^. This confirmed that the TiO_2_ particles were immobilized on the surface of the zeolite in the formation of nano-size. According to the BET analysis, it can be deduced that more active sites would be provided to participate in the reaction and that the ZTC would possess the potential for high photocatalytic efficiency.

### 3.2. Evaluation of the Photocatalytic Efficiency

**Figure 7:**
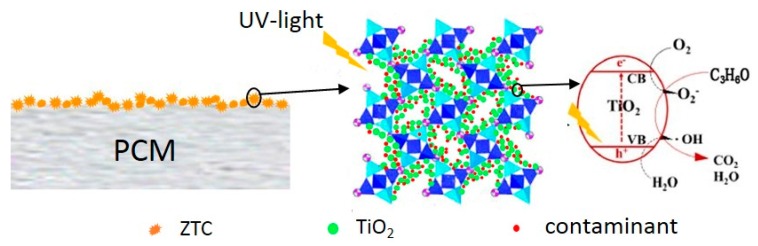
The photocatalysis mechanism of the PCM.

TiO_2_ is a kind of UV-light induced semiconductor catalyst. Upon the irradiation of the UV-light, the electron (e^−^) is excited from valance band to conduction band, thus leaving a hole (h^+^). The photo-induced electron-hole pairs can reach the surface of the TiO_2_ particle and start a reduction–oxidation process. The electron (e^−^) reacts with O_2_ to form superoxide anions ($O_{2}^{-}$), and the hole (h^+^) interacts with the hydroxyl (OH^−^) adsorbed on the surface of catalyst to generate hydroxyl radicals (**^•^**OH). The superoxide anions ($O_{2}^{-}$) and hydroxyl radicals (**^•^**OH) act as strong oxidants with the potential to decompose a wide range of organic contaminates [33,34]. Under the adequate operating conditions (residence time, UV irradiation), the final products should theoretically be carbon dioxide and water [30]. The photocatalysis mechanism of the PCM is shown in Figure 7. 

**Figure 8:**
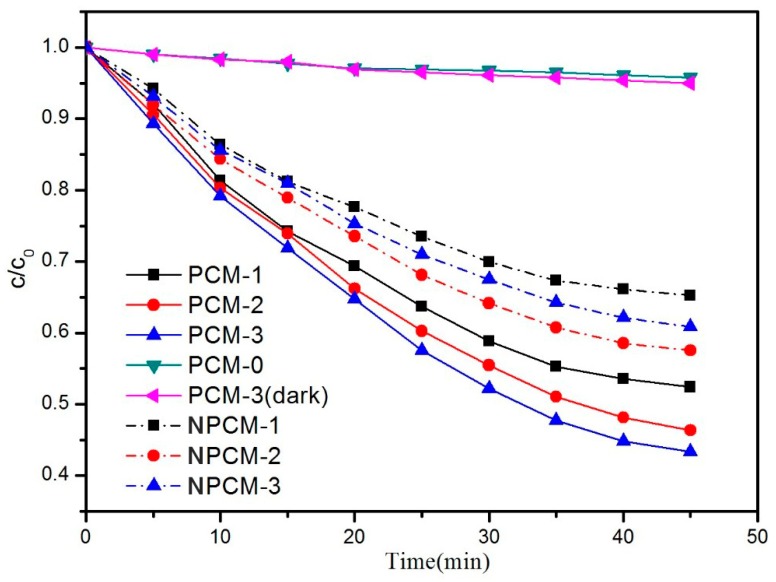
The degradation rate of acetone.

In Figure 8, the results of acetone removal are displayed. In the process of reaction, little gaseous intermediates were detected by GC-FID, which was in accordance with previous study [35]. The order for acetone removal rate is as below: PCM-3 > PCM-2 > PCM-1 > NPCM-2 > NPCM-3 > NPCM-1 > PCM-3(dark) > PCM-0. The results of PCM-3(dark) and PCM-0 suggested that the cement substrate could hardly decompose acetone without UV-light irradiation. For PCM-n (n = 1, 2, 3), the trend was obvious that the degradation efficiency increased with the growing of TiO_2_ content. Moreover, the acetone degradation rate of PCM-3 reached up to 56% which was remarkable, but the result cannot be compared with other previous reported studies, due to different experiment conditions and standards. The PCM-n (n = 1, 2, 3) samples had a higher photocatalytic efficiency than the corresponding NPCM-n (n = 1, 2, 3) samples. This was attributed to two effects. Firstly, the abundant channels and pores, and the large specific surface areas of zeolite benefitted to the gas diffusion and adsorption, correspondingly. Secondly, the zeolite was an optimal carrier for TiO_2_ due to the unique uniform pores, internal pore volume, and channel size [36,37,38], and TiO_2_ was proved to be homogeneously dispersed on the surface of the ZTC according to the results of N_2_ adsorption-desorption and SEM-EDS, thus more active sites were provided. The synergistic effect led to a high photocatalytic efficiency. The acetone removal rate of PCM-1 was higher than NPCM-n (n = 1, 2, 3), indicating that the ZTC enhanced the photocatalytic efficiency of the PCM with a smaller consumption of TiO_2_. The middle-carrier method is effective to improve the photocatalytic performance of the PCM. 

**Figure 9:**
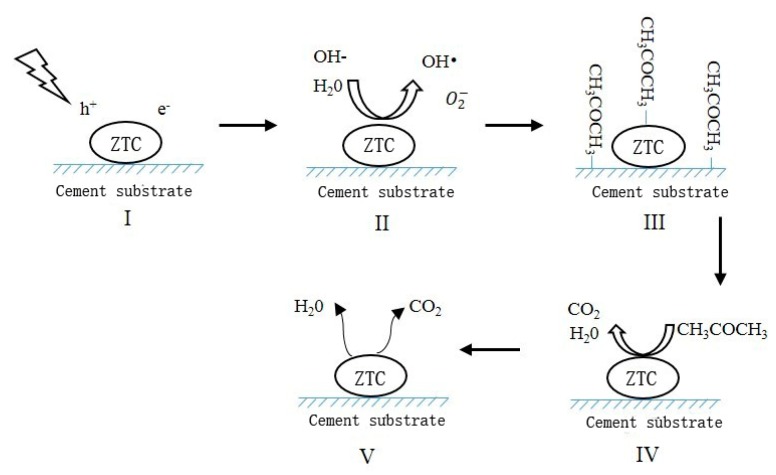
The kinetic processes of the degradation of acetone.

**Figure 10:**
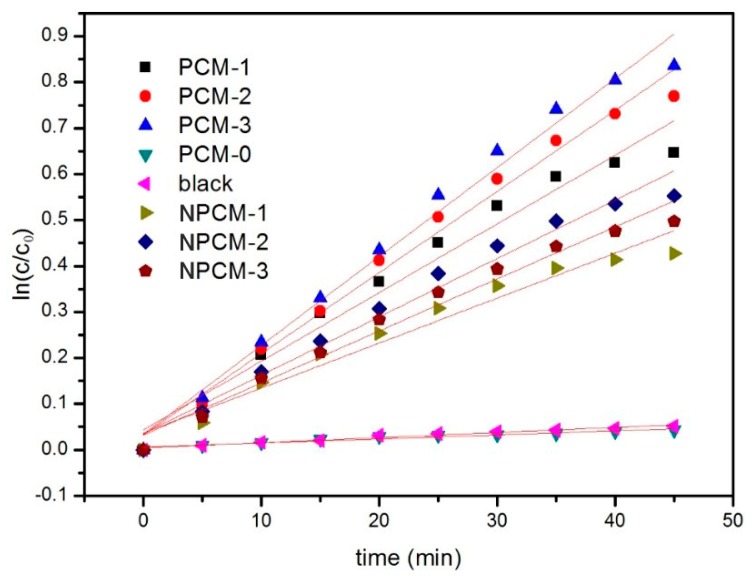
Photocatalytic reaction rates of acetone using different samples.

**Figure 11:**
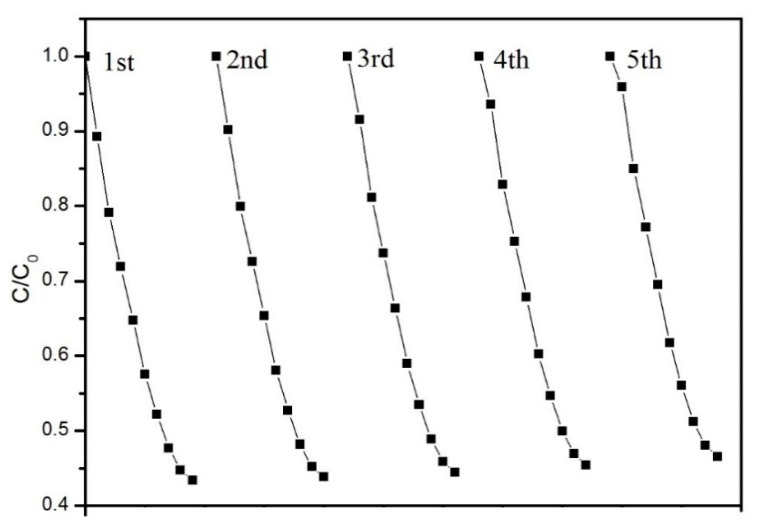
The cycling degradation rate of acetone by PCM-3.

According to the previous researches [8,9,39,40,41,42], the kinetic processes of acetone degradation is described in Figure 9. The photodegradation data of Figure 8 were fitted by using the first order kinetic model, and the results are shown in Figure 10. It was found that all the reactions were approximate to the first order reaction, and this was in accordance with previous study [43]. The reaction rate constants are shown in Table 6. The reaction rate changed along with the content of the ZTC. To our best knowledge, the reaction rate (k) is proportional to the concentration of the reactant under identical conditions. However, the initial concentration of acetone and H_2_O for all samples were constant. The most possible reason was that more $O_{2}^{-}$ and **^•^**OH were generated with the increasing content of ZTC, thus leading to higher reaction rate. It was obvious that the reaction rates of PCM-n were about 1.5 times higher than NPCM-n on average. It demonstrated that the special structure of the ZTC led to more active sites to improve the photocatalytic efficiency. As shown in Figure 11, the acetone removal rate of PCM-3 slightly decreased to 52% during the continuous test processes (5 times in total), and it was probably because some active sites were occupied by reaction products [44]. It can be inferred that the long-term photocatalytic performance of the PCM will be stable. 

## 4. Conclusions

(1) Due to the large specific surface area, natural zeolite is an optimal carrier for TiO_2_. After alkali corrosion and calcination, the surface of the MZ was suitable for a TiO_2_ precipitation. The TiO_2_ particles were homogeneously dispersed on the surface of the MZ in nano-scale, thereby providing a mass of active sites to participate in the photocatalytic reaction, leading to a high reaction rate.
(2) With the ZTC sprayed onto the surface of the cement matrix, the cement hydration products were not observed on TiO_2_ particles, indicating that the photocatalytic property of TiO_2_ particles would not be weakened by a cementitious system and the segregation protection of the ZTC was effective.
(3) Under the same test conditions, the acetone removal rate of PCM-3 reached up to 56% and the reaction rate of PCM-3 was 1.7 times higher than NPCM-3, proving that the ZTC had a high photocatalytic performance and that the middle-carrier method applied in this work contributed to the enhancement of the photocatalytic performance of the PCM.
(4) The removal efficiency of PCM-3 to the acetone was still 52% after cycling five times, thus confirming the durability and stability of the PCM.

## Figures and Tables

**Table 1:** The physical characteristics of natural zeolite.

Density (g/cm^3^)	Wear Rate	Pore Size (mm)	Water Absorption	Volume Weight (T/m^3^)
2.55	<0.03%	0.125~0.425	0.3%	1.70

**Table 2:** The chemical composition of natural zeolite (wt%).

SiO_2_	Al_2_O_3_	Na_2_O	CaO	K_2_O	MgO	Fe_2_O_3_	FeO	TiO_2_	P_2_O_5_
60~70	17.8	4.2	2.6	3.2	0.8	1.6	1.2	0.6	0.26

**Table 3:** The TiO_2_ content of ZTC-1, ZTC-2, and ZTC-3.

Specimen	TiO_2_-sol/MZ Ratio	TiO_2_ Content (wt%)
ZTC-1	0.47	0.99
ZTC-2	2.36	4.76
ZTC-3	4.72	9.10

**Table 4:** The composition of the photocatalytic cement-based material.

Specimen	Water (g)	Cement (g)	Photocatalyst Type	Photocatalyst (g)	TiO_2_ (g)
PCM-0	9.9	22	-	-	-
PCM-1	9.9	22	ZTC-1	0.220	0.002
PCM-2	9.9	22	ZTC-2	0.220	0.010
PCM-3	9.9	22	ZTC-3	0.220	0.020
NPCM-1	9.9	22	HTOP	0.002	0.002
NPCM-2	9.9	22	HTOP	0.010	0.010
NPCM-3	9.9	22	HTOP	0.020	0.020

**Table 5:** Specific surface area (S), Pore volume (V) and Average pore diameter (D) for MZ and ZTC-2.

Sample	S (m^2^g^−1^) ^a^	V (cm^3^g^−1^) ^b^	D (nm) ^c^
MZ	412	0.32	11.15
ZTC-2	319	0.24	10.87

^a^ Calculated with the BET method. ^b^ ^c^ Obtained from BJH desorption (1.9–300 nm).

**Table 6:** The reaction rates and relativity.

Specimen	Reaction Rates (k/min^−1^)	Relativity (R^2^)
blank	0.0011	0.9678
PCM-0	0.0008	0.9273
PCM-1	0.0140	0.9684
PCM-2	0.0177	0.9860
PCM-3	0.0194	0.9856
NPCM-1	0.0097	0.9581
NPCM-2	0.0120	0.9767
NPCM-3	0.0113	0.9766
